# Primary pulmonary adenocarcinoma mimicking papillary thyroid carcinoma

**DOI:** 10.1186/1749-8090-8-131

**Published:** 2013-05-17

**Authors:** Ya-Zhen Zhu, Wei-Ping Li, Zhi-Yuan Wang, Hai-Feng Yang, Qing-Lian He, Hong-Guang Zhu, Guang-Juan Zheng

**Affiliations:** 1 Department of Pathology, Guangdong provincial hospital of TCM, Guangzhou, University of Chinese medicine, Guangzhou, China; 2 Departments of Pathology, Shanghai Medical College, Fudan University, Shanghai, China; 3 Guangdong provincial academy of Chinese medical sciences, Guangzhou, China

**Keywords:** Pulmonary adenocarcinoma, Pulmonary papillary carcinoma, Papillary thyroid carcinoma

## Abstract

We herein reported a primary pulmonary papillary carcinoma with colloid-like luminal content in the glandular cavity and classic nuclear features such as pseudo-inclusions, intranuclear grooves in the tumor cell nuclei and ground glass nuclei which closely mimics papillary thyroid carcinoma. Meanwhile, lymph node in the left pulmonary hilum was involved and showed similar features to the primary pulmonary papillary carcinoma. This specific histopathological presentation caused a diagnostic dilemma.

The patient didn’t show previous concomitant or subsequent evidence of a thyroid tumor. Immunohistochemistry further confirmed pulmonary origin and excluded a metastasis from the thyroid, as it was thyroglobulin negative, thyroid transcription factor 1 and surfactant apoprotein A positive, which was consistent with the imageology and history.

Based on the above features, the diagnosis of primary pulmonary papillary carcinoma was confirmed. Understanding the existence of papillary thyroid carcinoma-like pulmonary papillary carcinoma will avoid misdiagnosis or unnecessary clinical and radiologic investigations in future.

## Background

Adenocarcinoma is the most common histologic subtype of lung cancer in most countries. Primary pulmonary adenocarcinomas are typically very heterogeneous, displaying a wide range of histologic features [1].

Primary pulmonary adenocarcinoma with enteric differentiation resembling metastatic colorectal carcinoma was first reported in 1991 as one particular type of adenocarcinomas, and enteric adenocarcinoma has been listed in the latest International Multidisciplinary classifications of lung tumors as one of the variants [2]. However, papillary thyroid carcinoma-like pulmonary papillary carcinoma has not yet been recognized as a histopathological variant. Only one such case has been previously reported that metastasis to the thyroid from lung adenocarcinoma could masquerade as thyroid carcinoma on fine needle aspiration (FNA) cytology [3], and that was a diagnostic pitfall in considering a metastasis tumor as a primary tumor. The case reported here represented a pitfall in the opposite direction. The current study reported an example of pulmonary papillary adenocarcinoma with colloid-like luminal content showed scalloped borders in the glandular cavity and classic nuclear features such as pseudo-inclusions, intranuclear grooves in the tumor cell nuclei, ground glass nuclei which closely mimics papillary thyroid carcinoma. To the authors’ knowledge, this is the first description of papillary thyroid carcinoma-like pulmonary papillary carcinoma based on resection specimen.

## Case presentation

An 83-year-old Chinese man, with a past history of hypertension for 12 years receiving regular treatment and coronary heart desease receiving Coronary artery stent implantation treatment 6 years ago, presented to our hospital complaining of anus mass prolapse after defecation for over 10 years. He had no history of malignancy, and no respiratory complaints were given during the review of systems. No abnormal breath sound was noted. The hemogram and blood chemistry were normal. During the regular examination before hemorrhoidectiomy, chest X-ray revealed a tubercular mass in the left lower lung field, then enhanced computed tomography (ECT) was performed and a nodular in the left lower pulmonary lobe with lymph node metastasis in the left pulmonary hilum was discovered. A whole body bone ECT scan was negative for skeletal metastasis.

The patient underwent left lower lobectomy to remove the mass and lymph node dissection as well as hemorrhoidectiomy. During the pathology diagnosis, ultrasonography was performed in the thyroid eliminating the diagnosis of primary thyroid carcinoma.

### Pathological findings

**Figure 1:**
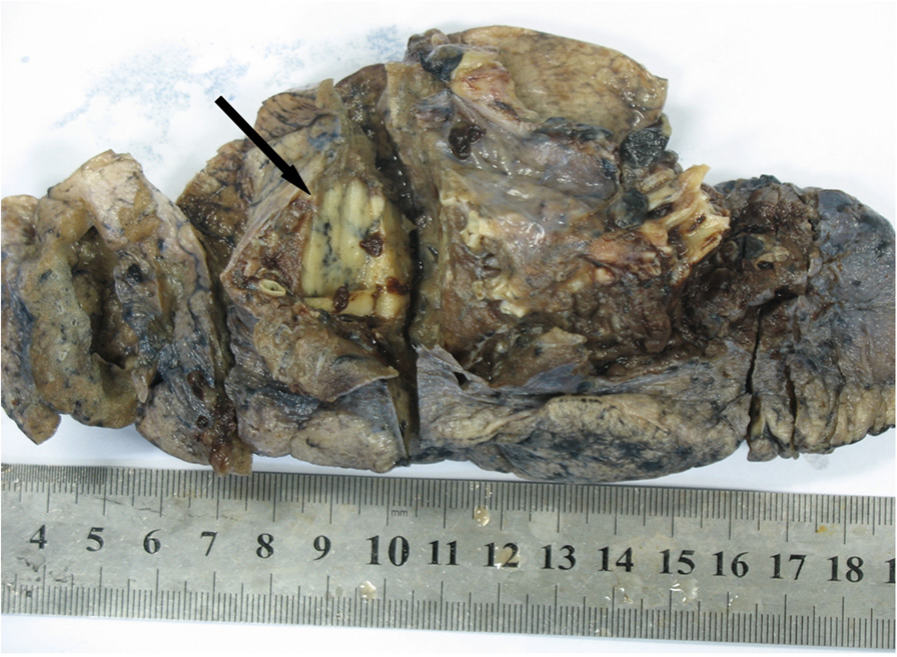
Macroscopic examination of the resected specimen of the left lower lobectomy showed a demarcated, white-gray, hard texture nodule, with 28 mm in diameter in the greatest axis.

Macroscopic examination of the resected specimen of the left lower lobectomy showed a demarcated, white-gray, hard texture nodule, with 28 mm in diameter in the greatest axis (Figure 1).

**Figure 2:**
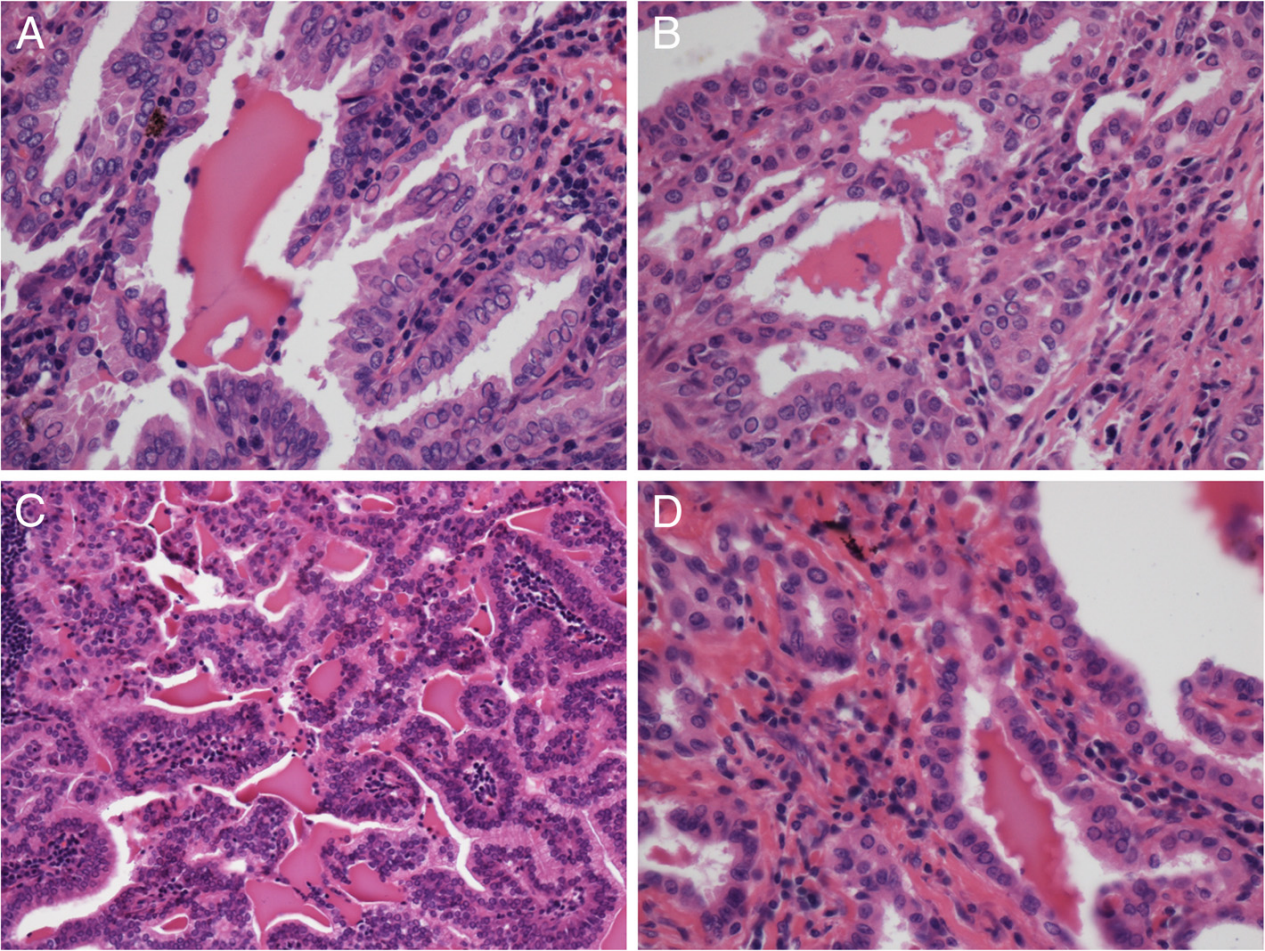
**Hematoxylin and eosin staining revealed that the eosinophilic and amorphous colloid-like luminal content showed scalloped border in the glandular cavity, pseudo-inclusions in the tumor cell nuclei or ground glass nuclei were evident in pulmonary tumor tissue (A**, **×400**; **B**, **×400) and metastasis lymph node (C**, **×200**; **D**, **×400).** The intranuclear groove in the tumor cell nuclei was evident in metastasis lymph node (**D**, ×400).

Histological examination revealed that the irregular glands in the pulmonary tumor tissue were composed of branching papillae with or without fibro-vascular cores, and the papillae was covered by a single to multiple layers of cuboidal to columnar cells. Eosinophilic pseudo-inclusions or intranuclear grooves in the tumor cell nuclei were seen in some area, and some colloid-like luminal content showed scalloped borders can be observed (Figure 2A, B).

In the tissue of metastasis lymph node in the left pulmonary hilum, the tumor presented well-differentiated branching papillae that mimicked papillary thyroid carcinoma with a lot of colloid-like luminal content showed scalloped borders in the glandular cavity, the nuclei of tumor cells contain finely dispersed chromatin, which imparts an optically clear or empty appearance, giving rise to the designation ground glass nuclei. In addition, invaginations of the cytoplasm may in cross-sections give the appearance of intranuclear inclusions ("pseudo-inclusions") or intranuclear grooves (Figure 2C, D). This combination of characteristics is typical of that seen in the papillary thyroid carcinoma.

**Figure 3:**
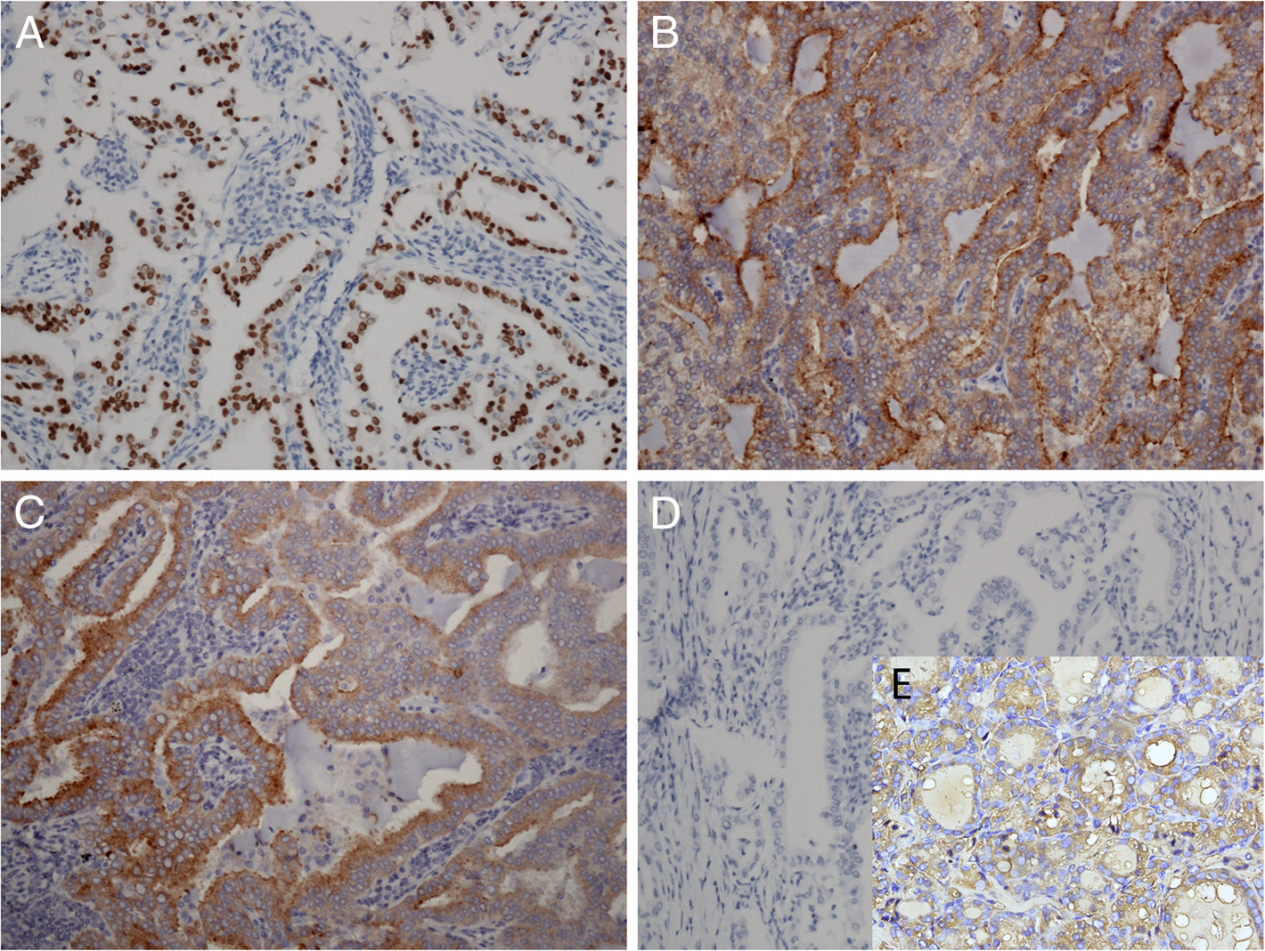
**Immunohistochemistry showed that TTF-1 was diffusely and strongly nuclear positive in pulmonary tumor tissue (A, ×200).** SPA was diffusely and strongly plasma positive in pulmonary tumor tissue (**B**, ×200) and lymph node (**C**, ×200). Thyroglobulin was negative in pulmonary tumor tissue (**D**, ×200). Thyroglobulin was positive in thyroid tissue as a positive control (**E**, ×200). TTF-1, thyroid transcriptor factor-1; SPA, surfactant apoprotein A.

Immunohistochemical analysis performed on paraffin sections revealed diffusely and strongly positive for surfactant apoprotein A (SPA), cytokeratin (CK), CK7, CK19, thyroid transcriptor factor-1 (TTF-1), focally and weakly positive for HBME-1, p63, and negative for TG in the pulmonary tumor tissue. Meanwhile, diffusely and strongly positive staining for SPA, CK19, focally and weakly positive staining for HBME-1, and negative staining for thyroglobulin (TG) in the lymph node in the left pulmonary hilum was demonstrated. Positive control samples reacted accordingly (Figure 3). The diluted primary antibody for SPA, CK, CK7, CK19, TTF-1, HBME-1, P63 and TG and the EnVision testing kit were purchased from QuanHui (GuangZhou, China).

Based on the above features, the diagnosis of primary pulmonary papillary carcinoma with lymph node metastasis in the left pulmonary hilum was confirmed. Extensive clinicopathologic examination failed to reveal a primary lesion in the thyroid either at the time of diagnosis or later during the follow-up.

After surgery, the patient completed 4 cycles of adjuvant chemotherapy. 7 months later, the examination showed recurrence of the tumors in both the left and right lung. The patient died 6 months later. No autopsy was performed.

## Discussion

Pulmonary adenocarcinoma is the most common, and the most diverse, heterogeneous form of primary lung carcinoma, the histological complexity of these tumors poses problems for pathologists [4]. It has been previously noted that some of the pure papillary carcinomas of the lung can easily mimic a papillary thyroid carcinoma [5]. Nuclear pseudoinclusions, although not totally specific, they are particularly common in papillary thyroid carcinoma, meningioma, and usual ductal hyperplasia of the breast and hence may aid in the diagnosis of these entities [6]. Therefore, the papillary carcinoma showing colloid-like luminal content in the glandular cavity and classic nuclear features such as pseudo-inclusions, intranuclear grooves in the tumor cell nuclei and ground glass nuclei raise immediately the possibility of a papillary thyroid carcinoma. However, if the tumor with these classic characteristics was found in the lung, it will evoke the possibility of a metastatic papillary thyroid carcinoma,since the lung is the most common site of systemic metastasis from thyroid carcinoma [7].

Generally, primary tumors are heterogeneous yet metastases are often homogeneous. Whenever unusual patterns of adenocarcinoma are encountered in the lung, consideration should always be given to the possibility of metastatic rather than primary disease [4]. However, the current case was specific because the histologic features of the primary pulmonary papillary carcinoma closely resembling those typical of the primary thyroid papillary carcinoma. In this case, negative TG staining and positive SPA staining should be helpful in the differential diagnosis, combined with the history study, palpation and ultrasonography in the thyroid.

Adenocarcinoma with thyroid differentiation resembling papillary thyroid carcinoma has been described in other tissue,such as breast. “Breast tumor resembling the tall cell variant of papillary thyroid carcinoma” was first reported in 2003 [8], after several more cases were accumulated, in 2012, some pathologists propose that we need to consider this lesion as a primary breast carcinoma and delete the words “thyroid carcinoma” from the terminology of this tumor. This approach will eliminate the need for further ancillary studies and reduce the unnecessary financial burden to the patient. This is particularly important when there is no clinical and/or imaging evidence of primary thyroid carcinoma [9]. Therefore, we believe that only by consistent, careful description of papillary thyroid carcinoma-like pulmonary papillary carcinomas will enough data be gathered from multiple centres to allow better understanding of their significance.

Pulmonary papillary carcinoma is a form of invasive adenocarcinoma. The significance of the papillary pattern is that it seems to represent relatively more aggressive, advanced, associated with metastatic risk and poorer survival [4]. Moreover, lymph node metastasis is one of the most important prognostic factors in pulmonary cancer. The present case showed lymph node metastasis in the left pulmonary hilum, but no skeletal metastasis was discovered by a whole body bone ECT scan. In this setting, left lower lobectomy and lymph node dissection was the surgical operation of choice.

## Conclusion

In summary, the histological characteristics in the current case are strongly reminiscent of a papillary thyroid carcinoma, the differential diagnosis may be virtually difficult without the help of immunohistochemistry. The current case represents a specific variant of primary pulmonary papillary carcinoma, although the diagnosis could be confirmed, understanding the existence of papillary thyroid carcinoma-like pulmonary papillary carcinoma will avoid misdiagnosis or unnecessary clinical and radiologic investigations in future, and accumulation of more cases are needed to clarify their significance.

## Consent

Written informed consent was obtained from the patient’s son for publication of this case report and any accompanying images. A copy of the written consent is available for review by the Editor-in-Chief of this journal.

## Competing interests

The authors declare that they have no competing interests.

## Authors’ contributions

Y-ZZ was a major contributor in writing the manuscript. Q-LH carried out the immunohistochemistry. W-PL, Z-YW and H-FY collected the clinical and pathological data, H-GZ and G-JZ conceived of the study, and participated in diagnosis and helped to draft the manuscript. All authors read and approved the final manuscript.

## References

[B1] Maeda R Isowa N Onuma H Pulmonary intestinal-type adenocarcinoma Interact Cardiovasc Thorac Surg 2008 7 349 351 18184677 10.1510/icvts.2007.168716

[B2] Travis WD Brambilla E Noguchi M International association for the study of lung cancer/american thoracic society/european respiratory society international multidisciplinary classification of lung adenocarcinoma J Thorac Oncol 2011 6 244 285 10.1097/JTO.0b013e318206a221 21252716 PMC4513953

[B3] Haraguchi S Hioki M Yamashita K Metastasis to the thyroid from lung adenocarcinoma mimicking thyroid carcinoma Jpn J Thorac Cardiovasc Surg 2004 52 353 356 10.1007/s11748-004-0070-7 15296034

[B4] Kerr KM Pulmonary adenocarcinomas: classification and reporting Histopathology 2009 54 12 27 10.1111/j.1365-2559.2008.03176.x 19187177

[B5] Moran CA Pulmonary adenocarcinoma: the expanding spectrum of histologic variants Arch Pathol Lab Med 2006 130 958 962 16831050 10.5858/2006-130-958-PATESO

[B6] Ip YT Dias Filho MA Chan JK Nuclear inclusions and pseudoinclusions: friends or foes of the surgical pathologist? Int J Surg Pathol 2010 18 465 481 21081532 10.1177/1066896910385342

[B7] Showalter TN Siegel BA Moley JF Prognostic factors in patients with well-differentiated thyroid cancer presenting with pulmonary metastasis Cancer Biother Radiopharm 2008 23 655 659 10.1089/cbr.2008.0501 18976119

[B8] Eusebi V Damiani S Ellis IO Breast tumor resembling the tall cell variant of papillary thyroid carcinoma: report of 5 cases Am J Surg Pathol 2003 27 1114 1118 10.1097/00000478-200308000-00008 12883243

[B9] Masood S Davis C Kubik MJ Changing the term "breast tumor resembling the tall cell variant of papillary thyroid carcinoma" to "tall cell variant of papillary breast carcinoma" Adv Anat Pathol 2012 19 108 110 10.1097/PAP.0b013e318249d090 22313838

